# Population Genetics of Pharmacogenetic Variants in a Greek Psychiatric Cohort of over 3000 Individuals

**DOI:** 10.3390/ijms26209896

**Published:** 2025-10-11

**Authors:** Eleni Ntoumou, Sevastiani Papailia, Dimitrios Miltiadis Vrachnos, Thanasis Fotis, Effie Salata, Angeliki Kapellou, Spiros Vittas

**Affiliations:** iDNA Laboratories, 7 Kavalieratou Taki, 14564 Kifissia, Greece; eleni.ntoumou@idna.gr (E.N.); sevastiani.papailia@idna.gr (S.P.); dimitris.vrachnos@idna.gr (D.M.V.); athanasios.fotis@idna.gr (T.F.); effie.salata@idna.gr (E.S.); angie.kapellou@idna.gr (A.K.)

**Keywords:** psychiatry, precision psychiatry, central nervous system, cytochromes, drug metabolism, response, precision medicine and personalized therapy, population pharmacogenetics/pharmacogenomics

## Abstract

Psychiatric disorders affect nearly one billion people worldwide and remain a major therapeutic challenge due to frequent treatment resistance. Pharmacogenetics provides a precision-informed approach by accounting for interindividual variability in drug metabolism and response, and population-specific data offer valuable information for therapeutic considerations. This study analyzed 3011 Greek individuals to assess 24 pharmacogenetic variants across 13 genes. Genotyping was performed using TaqMan OpenArray^®^ assays, and metabolic phenotypes were predicted based on established genotype-to-phenotype translation guidelines. Allele frequencies were compared with those in European, African, and East Asian populations. Population structure and genetic differentiation were evaluated using Principal Component Analysis (PCA), K-means clustering, fixation index (F_ST_), and STRUCTURE analysis. Results indicated that most allele frequencies in Greeks aligned with those in European populations, while several *CYP2D6* and *CYP2C19* variants differed significantly from those in African and East Asian cohorts. PCA and clustering confirmed strong European affinity, supported by low F_ST_ values, whereas STRUCTURE revealed minimal non-European admixture. Predicted metabolic phenotypes showed that 36%, 57.7%, and 41.6% of individuals exhibited altered CYP2D6, CYP2C19, and CYP2C9 activity, respectively. These findings highlight clinically actionable variation in the Greek population and emphasize the use of population-specific pharmacogenetic data to inform optimized strategies in precision psychiatry.

## 1. Introduction

Psychiatric disorders are affecting nearly 1 billion people and contributing significantly to global disease burden [1,2]. Among them, Major Depressive Disorder (MDD), Bipolar Disorder (BD) and Schizophrenia are the most severe and chronic conditions, often associated with significant functional impairment and reduced quality of life. Optimizing therapeutic outcomes remains a substantial challenge in psychiatric care. Around 30% of patients with MDD and 25% of BD do not achieve remission [3,4]. In Schizophrenia, approximately one third of patients are considered treatment-resistant, particularly to first-line antipsychotics [5]. These conditions present significant challenges, highlighting the urgent need for personalized strategies.

Pharmacogenetics (PGx) is a key aspect of precision medicine, providing valuable insights into interindividual variability in drug response and metabolism through genetic variants. The cytochrome P450 enzymes CYP2D6, CYP2C19, and CYP2C9 are responsible for the metabolism of a wide range of psychotropic drugs [6]. These enzymes can exhibit significant genetic diversity to common functional polymorphisms, which can alter enzyme activity and consequently, drug metabolism, resulting in metabolizer phenotypes classified as poor, intermediate, normal, or rapid and ultrarapid [6,7,8]. For instance, the CYP2D6*4 allele produces a non-functional enzyme and is associated with impaired metabolism of tricyclic antidepressants, increasing plasma concentrations and the risk of adverse effects. Recent studies further emphasize that *CYP2D6* and *CYP2C19* polymorphisms significantly impact antidepressant and antipsychotic drug exposure and clinical outcomes, supporting genotype-based dosing recommendations to improve treatment safety and efficacy [9,10]. To support clinical application, genotype-based prescribing guidelines have been developed by expert groups such as the Clinical Pharmacogenetics Implementation Consortium (CPIC) and the Dutch Pharmacogenetics Working Group (DPWG) [11]. The Association for Molecular Pathology (AMP) Group has established consensus guidelines aimed at standardizing the selection of genetic variants for clinical pharmacogenetic testing [12,13]. The evidence-based recommendations guide dose selection or alternative therapies based on metabolic phenotype, improving therapeutic outcomes and minimizing adverse drug reactions [11,12,13,14,15].

Several studies have shown that genetic polymorphisms affecting drug metabolism exhibit substantial interethnic variability, which results in potential critical implications for the implementation of pharmacogenetics in clinical practice [16]. Allele frequencies of key pharmacogenes such as *CYP2C19* differ markedly across different populations. For example, the CYP2C19*17 allele, which is associated with increased enzymatic activity, is more prevalent among European and African populations—ranging from 10% to 33%—compared to East Asians [10,16,17,18,19]. As a result, up to 33% of individuals in Europe and Africa are classified as ultrarapid metabolizers, whereas the corresponding prevalence in East Asia is around 2% [16,20]. This disparity is influencing the metabolic capacity for numerous psychotropic medications with potential consequences for drug efficacy and safety among different populations [16,21]. Therefore, elucidating the ethnogeographic distribution of pharmacogenetic variants is essential for improving risk stratification and optimizing therapy [22]. This highlights the importance of utilizing genetic diversity through population pharmacogenetics, as it facilitates the identification of variants specific to certain populations and supports the development of ethnically or geographically targeted recommendations based on the most prevalent alleles, thereby enhancing the accuracy of individual stratification [23,24].

Despite this genetic diversity within Europe, pharmacogenetic data from large, adequately powered cohorts of the Greek population remain limited, with most studies involving relatively small sample sizes [19]. This gap is particularly significant given Greece’s heterogeneous ethnic composition and strategic geographic position, highlighting the critical need for larger and more representative cohorts.

In the context of psychiatric practice in Greece, incorporating population-specific pharmacogenetic data offers significant potential to optimize drug selection and dosing, reduce adverse drug reactions, and improve therapeutic outcomes. Building on recent advances in pharmacogenetics, this study aims to analyze allele frequencies of 24 key single-nucleotide polymorphisms (SNPs) in 13 genes in patient-derived DNA samples from a Greek population and determine the prevalence of potential clinically actionable pharmacogenetic phenotypes to inform the application of precision-guided psychiatric therapeutics. Additionally, this study aims to investigate the associations between variations in the allele frequencies of the 24 SNPs across different populations and the subsequent pharmacogenetic diversity. By comparing allele frequencies, population structure, and differentiation metrics between Greek individuals and major reference populations (European, African, and East Asian), this approach will enable to investigate pharmacogenetic variants from a PGx panel that exhibit significant divergence across populations.

## 2. Results

### 2.1. Population Characteristics

**Figure 1:**
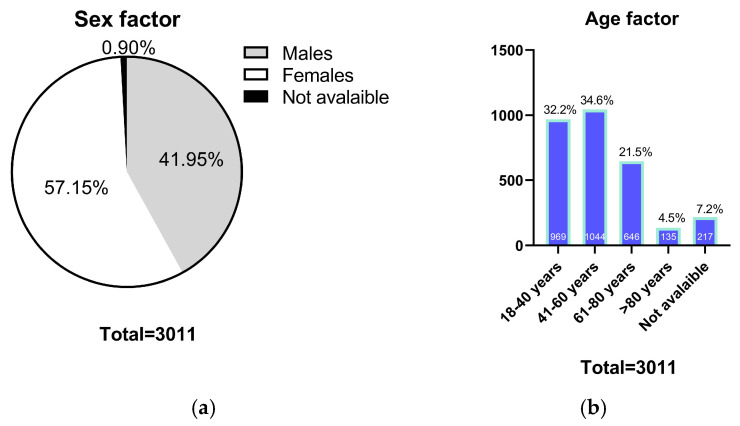
(**a**) Sex distribution of the study cohort. Pie chart illustrating the proportion of female and male participants among the 3011 individuals included in the study; (**b**) Age distribution of the study cohort. Pie chart showing the distribution of participants across five age groups: 18–40 years, 41–60 years, 61–80 years, and over 80 years.

A total of 3011 samples were collected from a Greek psychiatric cohort. The study population consisted of 1720 females (57.15%) and 1263 males (41.95%) (Figure 1a). The age distribution of the cohort was as follows: 969 samples (32.18%) from individuals aged 18–40 years, 1044 samples (34.67%) aged 41–60 years, 646 samples (21.45%) aged 61–80 years, and 135 samples (4.48%) from individuals aged over 80 years (Figure 1b).

### 2.2. Pharmacogenetic Variant Frequencies Distributions Between Greek and Other Continental Populations

Allele frequencies of 24 clinically relevant pharmacogenetic SNPs were analyzed in a psychiatric cohort of 3011 Greek individuals using the PGx-CNS panel. This study provides, for the first time, allele frequency data for these specific SNPs in a large Greek cohort (*n* = 3011). To assess population-specific variation, allele frequencies in the Greek cohort were compared to publicly available data from three reference populations: European (EUR), African (AFR), and East Asian (EAS).

Allele frequencies for most variants studied in the Greek cohort showed consistency with those observed in the European cohort, with no significant differences (*p* > 0.01 after Holm-Bonferroni correction) for many SNPs such as rs1799853 (*CYP2C9*), rs1414334 (*CYP2D6*), and rs4244285 (*CYP2C19*). However, several SNPs exhibited significant allele frequency differences when compared to the AFR and EAS cohorts, including rs1065852, rs28371725, rs35742686, and rs3892097 within the *CYP2D6* gene. Among the 24 SNPs analyzed, only rs28399504 in the *CYP2C19* gene exhibited similar allele frequencies across all four populations, indicating a conserved distribution. The A allele of rs4986893 (*CYP2C19*), a rare variant was detected at very low frequencies in the Greek cohort, consistent with European ancestry, and in contrast with the AFR and EAS cohorts where it appeared more prevalent.

These detailed allele frequency distributions and interpopulation comparisons are summarized in Table 1, which presents allele frequencies in the Greek cohort alongside corresponding frequencies and statistical comparisons with the EUR, AFR, and EAS populations.

### 2.3. Population Structure Analysis Through PCA and K-Means Clustering of PGx Variants

To evaluate the capacity of the iDNA PGx-CNS panel to resolve population structure and assess genetic similarity between the Greek population and global reference groups, we performed PCA and unsupervised clustering based on pharmacogenetic variant data. The dataset included Greek individuals (*n* = 500) alongside reference populations from the 1000 Genomes Project: EUR (*n* = 503), AFR (*n* = 662), and EAS (*n* = 504).

**Figure 2:**
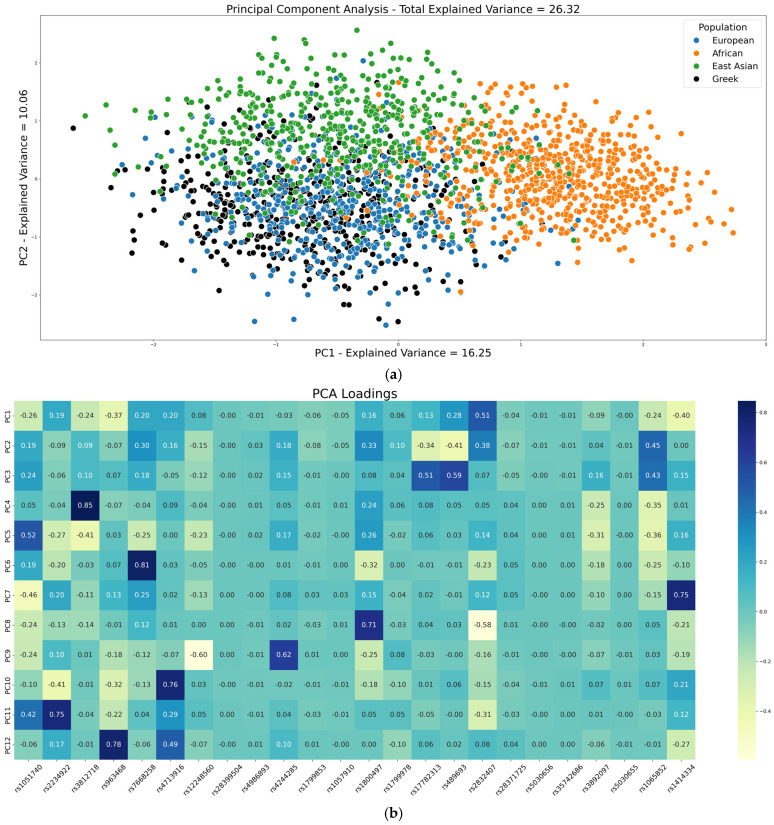
(**a**) PCA of the Greek and Global Populations Based on Pharmacogenetic variants. A scatter plot of the first two principal components (PC1 and PC2) displays the genetic structure of the Greek population (black) in comparison to global reference populations from the 1000 Genomes Project: EUR (blue), AFR (orange), and EAS (green); (**b**) Heatmap depicting the contribution of individual SNPs to principal components in the population genetic analysis. The color gradient ranges from light yellow to dark blue, corresponding to impact values from 0 (minimal contribution) to 1 (maximal contribution), thereby highlighting loci with the greatest influence on population structure and differentiation.

Comparison of the first two principal components demonstrated that PC1 primarily captured genetic differentiation between the AFR population and the EUR and EAS populations, while PC2 reflected genetic variance distinguishing EUR from EAS groups. These patterns are consistent with expected continental-scale population structure shaped by historical divergence. Greek individuals exhibited clustering patterns strongly consistent with the EUR population, showing substantial genetic affinity, although some degree of overlap with neighboring clusters was observed, reflecting admixture and shared ancestral polymorphisms (Figure 2).

To further characterize population structure, K-means clustering was applied to the first two principal components. At *K* = 3, nearly all Greek individuals clustered with the EUR reference group. This clustering solution yielded a silhouette score of 0.13, marginally higher than the 0.12 observed at *K* = 4, indicating moderately coherent clustering with three genetic clusters.

**Figure 3:**
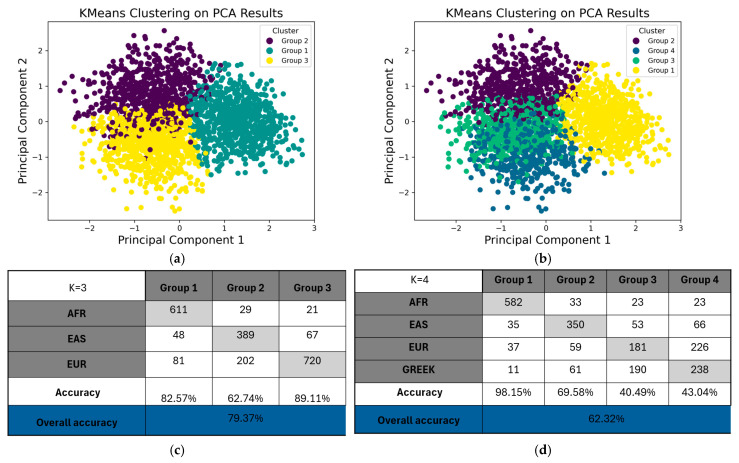
K-means Clustering of Individuals Based on PC1 and PC2 From Pharmacogenetic PCA: K-means clustering was applied to the first two principal components (PC1 and PC2) derived from pharmacogenetic variant data to identify genetic subgroups. (**a**) Clustering results for *K* = 3; (**b**) Clustering results for *K* = 4; (**c**) Correspondence between inferred PGx clusters (*K* = 3) and global reference groups (EUR, AFR, EAS); (**d**) Correspondence between inferred PGx clusters (*K* = 4) and global reference groups (EUR, AFR, EAS).

Clustering performance metrics were similar between both clustering schemes, with an Adjusted Rand Index (ARI) of 0.42 and Normalized Mutual Information (NMI) of 0.39 for *K* = 3, and ARI = 0.401 and NMI = 0.40 for *K* = 4. These results (Figure 3) indicate that the iDNA PGx-CNS panel captures broad continental genetic structure, consistent with known patterns of population differentiation and admixture, allowing confident assignment of Greek individuals within the European genetic background.

### 2.4. Assessment of Population Divergence Between Greeks and Global Populations Based on the F_ST_ and STRUCTURE

**Figure 4:**
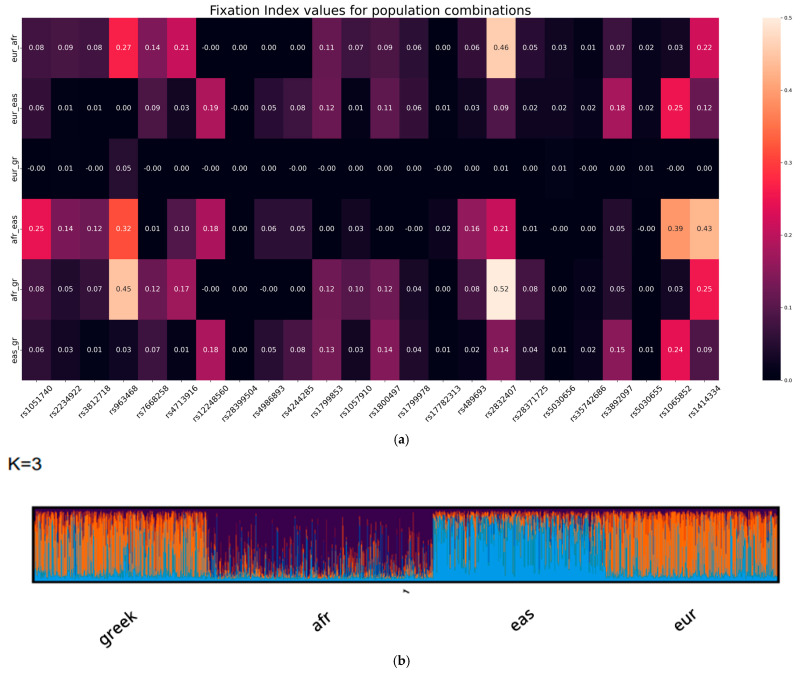
(**a**) Heatmap of genetic differentiation (F_ST_ values) between the Greek population and continental reference groups across pharmacogenetic variants. The heatmap displays pairwise F_ST_ values ranging from 0 to 0.5, quantifying genetic differentiation between the Greek population and three major continental populations: EUR, AFR, and EAS; (**b**) CLUMPAK bar plots from STRUCTURE. Each vertical bar along the *x*-axis represents a single individual, with colors indicating inferred ancestry components. The results reflect genetic admixture patterns across the analyzed populations.

Population-level differentiation observed through PCA and clustering analysis was further supported by F_ST_ values calculated across the pharmacogenetic variant panel. The F_ST_ quantifies genetic differentiation between populations, with values ranging from 0 (no differentiation) to 1 (complete differentiation). Genetic differentiation between the Greek and broader EUR populations was minimal, with low F_ST_ values (<0.02) across most loci, indicating a high level of genetic homogeneity. In contrast, moderate to high levels of genetic divergence were observed between the Greek and AFR populations, particularly at three key loci—rs2832407, rs963468, and rs1414334—with F_ST_ values ranging from 0.25 to 0.52 (Figure 4a). These variants also showed high loadings on PC1, contributing substantially to the genetic differentiation of AFR individuals from the Greek–European cluster.

Between the Greek and EAS populations, moderate differentiation was captured by F_ST_ values at several clinically actionable loci, including rs1065852 (F_ST_ = 0.24), rs12248560 (F_ST_ = 0.18), and rs3892097 (F_ST_ = 0.15) (Figure 4a). These variants contributed notably to PC2 in the PCA, supporting the genetic distinction observed between the EAS and Greek–European clusters. Consistent with the PCA results, no significant population structure was detected between the Greek and other European samples based on F_ST_ values.

STRUCTURE, a Bayesian model-based clustering algorithm, was employed to analyze individual genetic ancestry proportions based on 24 pharmacogenetically informative SNPs. The analysis included 1699 individuals from the 1000 Genomes Project and the Greek population (*n* = 500). The high genetic proximity observed between the Greek and broader (EUR) populations using this SNP panel warranted the selection of a model with *K* = 3 ancestral populations to represent major continental groups while reflecting limited differentiation between closely related populations.

The results showed distinct ancestry components corresponding to each continental group, with notable admixture patterns. Greek and EUR individuals exhibited highly similar ancestry profiles, with limited contribution from EAS-related components, consistent with findings from PCA and population differentiation analyses. The AFR population was clearly differentiated. Mean divergence values for each inferred cluster supported these patterns: the AFR cluster showed the lowest divergence (0.1070), the EUR-Greek cluster exhibited the highest differentiation (0.4024), and the EAS cluster demonstrated intermediate divergence (0.2121), consistent with global patterns of population structure and genetic drift (Figure 4b).

### 2.5. Clinically Actionable Variants

To assess the prevalence of predicted clinically actionable pharmacogenetic phenotypes based on the available SNP PGx panel, individuals were classified into predicted metabolizer categories for the cytochrome P450 enzymes CYP2D6, CYP2C19, and CYP2C9, revealing considerable interindividual variability within the Greek cohort. Predicted phenotypic categories included normal, ultra-rapid, rapid, intermediate, and poor metabolizers.

**Figure 5:**
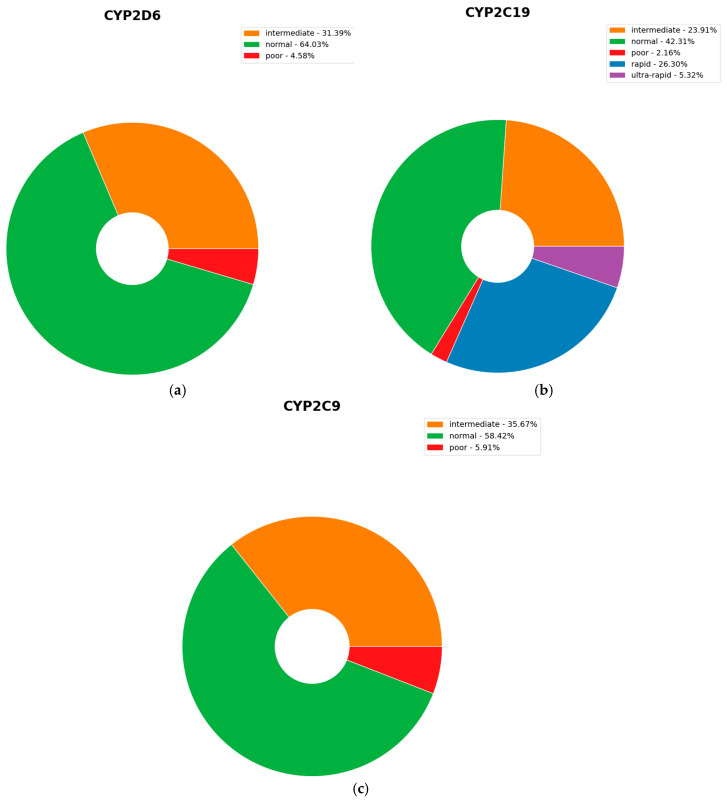
(**a**) Percentage of each CYP2D6 predicted metabolizer phenotype; (**b**) Percentage of each CYP2C19 predicted metabolizer phenotype; (**c**) Percentage of each CYP2C9 predicted metabolizer phenotype.

For CYP2D6, 64.03% of individuals were predicted as normal metabolizers, 31.39% as intermediate, and 4.58% as poor metabolizers, indicating that approximately 36% of the population exhibited altered CYP2D6 metabolism (intermediate or poor). For CYP2C19, 42.31% were predicted as normal metabolizers, 23.91% intermediate, 2.16% poor, 26.30% rapid, and 5.32% ultra-rapid metabolizers. For CYP2C9, 58.42% were predicted as normal, 35.67% intermediate, and 5.91% poor metabolizers, showing that 41.6% of individuals exhibited altered CYP2C9 metabolic activity (Figure 5).

It is important to note that these phenotype predictions are based solely on the alleles included in the available SNP PGx panel, which may limit the accuracy of diplotype-to-phenotype conversions and the reported metabolizer frequencies. Overall, while this analysis provides valuable insight into predicted metabolic phenotypes within the Greek cohort, these limitations are clearly acknowledged to promote transparency and guide future improvements in panel design and variant coverage.

Overall, the findings revealed a high degree of genetic similarity to European populations, with notable differences from African and East Asian groups. Notably, a substantial proportion of individuals in the Greek population exhibited altered predicted metabolizer status for CYP2D6, CYP2C19, and CYP2C9.

## 3. Discussion

Pharmacogenetic variation related to CNS drugs was examined in a cohort of 3011 Greek individuals using the iDNA PGx-CNS panel. This study presents the first large-scale analysis of clinically actionable pharmacogenetic variants related to CNS drug response in the Greek population The findings reveal that Greeks are genetically aligned with European populations while exhibiting differences from African and East Asian groups. Importantly, a substantial proportion of individuals exhibited altered CYP2D6, CYP2C19, and CYP2C9 predicted metabolizer phenotypes, highlighting the translational relevance of integrating population-specific pharmacogenetic data into personalized psychiatric therapy.

Allele frequencies for 24 pharmacogenetic SNPs, including rs1799853 (*CYP2C9*), rs1414334 (*CYP2D6*), and rs4244285 (*CYP2C19*) were broadly concordant with those reported in European cohorts. In contrast, several SNPs —particularly within *CYP2D6* displayed notable differences compared to African and East Asian populations, highlighting interethnic variability consistent with prior studies emphasizing population differences in drug-metabolizing genes [25,26]. One exception was rs28399504 (*CYP2C19*), which showed consistent frequencies across all groups, suggesting evolutionary conservation. The low frequency of rare alleles such as rs4986893 aligns with the pattern observed in European populations. These findings corroborate prior investigations on a smaller cohort of 501 individuals, confirming the necessity of larger and more representative cohorts for robust investigation of pharmacogenetic variation [27].

The observed ethno-geographic variability in pharmacogenetic alleles highlights the importance of inclusive research encompassing diverse and admixed populations to avoid biased clinical decision-making and to support equitable precision medicine initiatives. Accurate ancestry reporting and broadened population studies remain critical to this effort [24]. In the future, integrating population-specific data into clinical algorithms will be crucial not only for reducing inequities but also for creating adaptive PGx guidelines that evolve alongside global demographic shifts and migration patterns.

Accordingly, population structure analyses via PCA and unsupervised clustering demonstrated clear continental genetic stratification, with Greek individuals clustering tightly within European reference populations, consistent with their genetic homogeneity. Moderate to high F_ST_ values distinguishing Greeks from African and East Asian populations at key pharmacogenetic loci reinforced these findings. Bayesian clustering analysis using STRUCTURE also identified distinct ancestry components corresponding to major continental groups, with Greek and European individuals exhibiting highly similar profiles and minimal admixture from other ancestries. These results align with other studies leveraging limited but informative pharmacogenetic panels to resolve major population genetic structure, such as a recent study demonstrating that PGx SNP panels accurately estimate individual genetic ancestry (IGA) and enable the development of population-specific clinical decision support tools, including an African American–specific warfarin dosing algorithm with high sensitivity and specificity [28,29]. This underscores the potential of targeted pharmacogenetic approaches to inform personalized medicine across diverse populations. Future research could explore whether PGx panels for psychiatric drugs provide similar predictive utility for ancestry inference, thereby linking population genetics with actionable therapeutic recommendations.

The genetic homogeneity of Greeks with Europeans [30] supports the clinical application of established PGx guidelines, such as those from the CPIC [31], are broadly applicable. Nevertheless, ongoing local data acquisition is important to refine clinical algorithms and minimize unexpected variability in drug response. Further, the pronounced interpopulation variability in *CYP2D6* alleles, notably the higher frequency of functional and duplicated alleles linked to ultrarapid metabolizer phenotypes in African populations, highlights the necessity for population-specific PGx guidance [32]. Such genetic differences bear direct clinical relevance, influencing dosing and response to critical medications, guiding which pharmacogenetic tests should be prioritized for cost-effectiveness, and shaping dosing guidelines and regulatory recommendations tailored to specific populations, thereby supporting safer and more effective therapeutic strategies.

Phenotypic prediction revealed significant interindividual variation in metabolic capacity for CYP2D6, CYP2C19, and CYP2C9 within the Greek cohort. Approximately one-third of the cohort (36%) exhibited altered CYP2D6 metabolism, predominantly as intermediate metabolizers (31.38%), with a smaller proportion classified as poor metabolizers (4.58%). Altered CYP2C19 activity was more prevalent, affecting over half of individuals (57.7%), encompassing decreased function (intermediate 23.91%, poor 2.16%) as well as increased function (rapid 26.3%, ultra-rapid 5.31%). Altered CYP2C9 metabolism was observed in 41.6% of participants, mainly as intermediate metabolizers (35.67%) and to a lesser extent as poor metabolizers (5.91%). These predicted phenotypic frequencies are based only on the alleles included in the PGx-CNS panel and should be interpreted within this framework. These findings are consistent with prior work in the Greek population, [27] further supporting the clinical utility of integrating pharmacogenetic data to optimize therapeutic outcomes. This considerable predicted prevalence of altered metabolizer phenotypes highlights the importance of pharmacogenetic testing in guiding individualized treatment strategies, particularly for CNS drugs, where dosing, efficacy, and adverse event profiles can be substantially influenced by metabolic variability. For example, poor metabolizers, of CYP2D6 have reduced metabolism of clomipramine and increased risk of side effects. Pharmacogenetic guidance recommends reduction in the initial recommended dosage of clomipramine [33].

In summary, this large-scale study provides compelling evidence supporting the integration of population-stratified pharmacogenetic data into precision CNS therapeutics. The findings confirm the close genetic similarity of Greeks to European population while showing notable divergence from African and East Asian groups. In parallel, the high prevalence and marked interindividual variability of altered metabolizer phenotypes in CYP2D6, CYP2C19, and CYP2C9 underscore the critical clinical value of pharmacogenetic testing to tailor treatment, improve drug safety, and enhance efficacy. These results highlight the importance of ancestry-informed approaches and population-specific data to guide personalized CNS drug regimens. Ongoing local data collection and research remain essential to refine clinical algorithms, overcome implementation challenges, and promote equitable access to optimized CNS pharmacotherapies across diverse patient populations [34]. Ultimately, the incorporation of pharmacogenetic testing into psychiatric practice has the potential to move the field closer to precision psychiatry, reducing trial-and-error prescribing and improving outcomes for patients facing the burden of treatment-resistant mental illness.

## 4. Materials and Methods

### 4.1. Samples and Data

This study included a cohort of 3011 participants from Greece who were tested using a pharmacogenetic panel (iDNA PGx-CNS) between February 2020 and 31 December 2023. Inclusion criteria encompassed adults of any age and sex who were referred by a psychiatrist. The researchers did not have access to participants’ clinical diagnoses. Exclusion criteria applied to cases who did not provide informed consent. All participants provided informed consent, allowing the use of their anonymized genetic data for research and statistical purposes. The study was conducted according to Helsinki declaration principles. Formal ethical committee approval was not required due to the retrospective nature of the study. Buccal swab samples were collected from Greek individuals using the iDNA PGx-CNS kit (iDNA Laboratories SA, Athens, Greece).

Genomic DNA was extracted from 300 μL of phosphate-buffered saline (PBS) in which the buccal cells were diluted, using the PureLink Genomic DNA Mini Kit (Invitrogen, Thermo Fisher Scientific, Waltham, MA, USA). The quality and quantity of the extracted DNA were assessed using the NanoDrop One Spectrophotometer (Thermo Fisher Scientific, Waltham, MA, USA). Samples were required to have a DNA concentration of 20 ng/μL, an A260/A280 ratio of approximately 1.8, and an A260/A230 ratio within the range of 2.0–2.2 to proceed with genotyping. Until further use, DNA samples were stored at −20 °C [35].

### 4.2. Genotyping

Genotyping was carried out with the TaqMan Custom OpenArray^®^ Genotyping Assays at the QuantStudio™ 12 K Flex Real-Time PCR System (Applied Biosystems by Life Technologies, Carlsbad, CA, USA), in accordance with the manufacturer’s protocol. The 24 targets (SNPs) under evaluation in this panel were incorporated in the custom OpenArray Genotyping plates (ThermoFischer Scientific, Waltham, MA, USA). The list of analyzed SNPs and associated genes is presented in Supplementary Materials Table S1. The PCRs were performed in a total volume of 6 μL, containing 3 μL of TaqMan™ OpenArray^®^ Genotyping Master Mix (ThermoFischer Scientific, Waltham, MA, USA) and 3 μL of genomic DNA with a total mass of 70 ng. At least one no-template control (NTC) that contained water was included per OpenArray^®^ plate, serving as a negative control and resulting in a no amplification signal. The analysis of the raw data obtained from the thermocycler was performed with QuantStudio™ 12 K Flex software v1.5 and TaqMan Genotyper software v1.3, using the method of allelic discrimination.

### 4.3. Comparative Analysis of Variant Frequencies Across Different Populations

#### 4.3.1. Statistical and Bioinformatic Analysis

Statistical analyses were conducted using R (version 4.2.2). For each of the 24 SNPs evaluated, the associations between population groups and genotype frequencies were tested using Pearson’s chi-square test. In instances where the assumptions of the chi-square test were not met—specifically, when any expected cell count was zero or when more than 80% of the expected counts were fewer than five—Fisher’s exact test of independence was applied. To control the family-wise error rate, multiple testing corrections were performed using the Holm-Bonferroni method [36].

Genotyping data for 24 SNPs on 13 genes were analyzed using the iDNA PGx–CNS bioinformatic algorithm, which automatically interprets pharmacogenenetic data as previously described [27]. Based on genotypic results, individuals were categorized into predicted metabolic phenotypes—normal, ultra-rapid, rapid, intermediate, or poor metabolizers—for the CYP2C9, CYP2C19, and CYP2D6 cytochrome P450 enzymes. The iDNA PGx panel used in this study does not cover all clinically relevant alleles recommended by the Association for Molecular Pathology (AMP), which may limit the accuracy of diplotype-to-phenotype conversions and the reported metabolizer frequencies. Comparative analyses of allele frequencies across populations were also performed using chi-square tests to identify statistically significant differences.

#### 4.3.2. Population Genetics Analysis

For all steps of the statistical analysis, Variant Call Format (VCF) files from phase 3 of the 1000 Genomes Project were downloaded. Data from the European (*n* = 503), African (*n* = 662), and East Asian (*n* = 504) populations were used, totaling 1669 individuals [35]. For the Greek population, we selected a random sample of 500 individuals from the original cohort of 3011, to match the population volume of the other three main populations (European, African, and East Asian). In order to infer genetic ancestry, PCA was performed to visualize genetic clustering [37,38,39].

To identify cluster definitions and distances we used the K-means algorithm for *K* = 3 and *K* = 4. Additionally, F_ST_ and Chi square tests were carried out. Python v3.12.11 packages Scikit-learn v1.7.0, SciPy v1.15.3 and Scikit-allel v1.3.13 were used for analysis. Matplotlib v3.10, and Seaborn v0.13.2 packages were used for graphs and diagrams. Finally, the clustering algorithm STRUCTURE v2.3.4 was used to estimate individual genetic ancestry and admixture proportions. The length of burning period was set to 10,000 and the Markov Chain Monte Carlo to 50,000. The algorithm was run for *K* = 3 and *K* = 4, 10 times for each and the CLUMPAK server (https://clumpak.tau.ac.il/, accessed on 21 November 2025) was used to visualize results [40,41,42,43,44].

## 5. Conclusions

The Greek population exhibited close genetic similarity to other European groups, while diverging significantly from African and East Asian populations. High prevalence of and variability in predicted altered metabolizer phenotypes in CYP2D6, CYP2C19, and CYP2C9 were observed, demonstrating their potential impact on treatment response, highlighting the value of pharmacogenetic guidance. This study bridges a crucial gap in precision psychiatry by presenting the largest pharmacogenetic analysis of Greek individuals to date. It demonstrates that population-stratified pharmacogenetic analysis can enhance precision therapeutics in psychiatric disorders. Future research should validate these findings in larger cohorts, refine clinical algorithms, and address remaining methodological challenges, including expanding the current PGx panel used in this study, which may improve phenotype assignment, ultimately facilitating broader implementation of pharmacogenetically guided therapies in psychiatry.

## Figures and Tables

**Table 1:** Genotype frequencies of the iDNA PGx–CNS panel and statistical results from the Chi-square pairwise comparisons of gene variant frequencies between the study sample (Greeks) and the following populations: EUR, AFR, and EAS. The asterisks (*) indicate the comparisons for which Fisher’s exact test of independence was performed. In bold are the *p*-values that remained significant (*p*-value < 0.01) after being corrected using the Holm-Bonferroni method.

Gene Variants	Allele_1	Frequencies (Greeks)	Allele_2	Frequencies (Greeks)	Greeks-EUR	Greeks-AFR	Greeks-EAS
rs1799853	C	85.6%	T	14.4%	>0.99	**<0.01**	**<0.01**
rs28371725	C	87.7%	T	12.3%	0.2	**<0.01**	**<0.01**
rs2832407	A	33.6%	C	66.4%	0.66	**<0.01**	**<0.01**
rs1414334	C	13.3%	G	86.7%	>0.99	**<0.01**	**<0.01**
rs1065852	A	20.0%	G	80.0%	>0.99	**<0.01**	**<0.01**
rs35742686	T	98.4%	-	1.6%	>0.99	**<0.01**	**<0.01**
rs28399504	A	99.5%	G	0.5%	>0.99 *	0.08	0.22 *
rs4244285	A	13.6%	G	86.4%	>0.99	**<0.01**	**<0.01**
rs5030656	CTTCT	99.1%	CT	0.9%	**<0.01**	0.02	0.02
rs1799978	C	7.8%	T	92.2%	0.84	**<0.01**	**<0.01**
rs4713916	A	28.4%	G	71.6%	0.66	**<0.01**	**<0.01**
rs5030655	A	99.3%	-	0.7%	**<0.01**	0.06	0.04
rs3892097	C	83.6%	T	16.4%	>0.99	**<0.01**	**<0.01**
rs4986893	G	99.9%	A	0.1%	>0.99 *	0.21 *	**<0.01**
rs2234922	A	79.3%	G	20.7%	0.04	**<0.01**	**<0.01**
rs1051740	C	29.3%	T	70.7%	>0.99	**<0.01**	**<0.01**
rs489693	A	30.7%	C	69.3%	>0.99	**<0.01**	**<0.01**
rs12248560	C	79.0%	T	21.0%	>0.99	0.09	**<0.01**
rs7668258	C	52.4%	T	47.6%	>0.99	**<0.01**	**<0.01**
rs3812718	C	48.5%	T	51.5%	>0.99	**<0.01**	**<0.01**
rs1057910	A	90.6%	C	9.4%	0.66	**<0.01**	**<0.01**
rs963468	A	37.5%	G	62.5%	>0.99	**<0.01**	0.27
rs1800497	A	16.6%	G	83.4%	>0.99	**<0.01**	**<0.01**
rs17782313	C	24.4%	T	75.6%	>0.99	0.06	**<0.01**

## Data Availability

The data presented in this study are available upon request from the corresponding author. The data are not publicly available due to restrictions imposed by the data owner, as they are the proprietary property of iDNA Laboratories S.A., Private Diagnostic Laboratories.

## Supplementary Materials

The following supporting information can be downloaded at: https://www.mdpi.com/article/10.3390/ijms26209896/s1.

## Abbreviations

The following abbreviations are used in this manuscript:

PGx	Pharmacogenetics
MDD	Major Depressive Disorder
BD	Bipolar Disorder
CPIC	Clinical Pharmacogenetics Implementation Consortium
DPWG	Dutch Pharmacogenetics Working Group
AMP	Association for Molecular Pathology
SNPs	Single-Nucleotide Polymorphisms
PBS	Phosphate-Buffered Saline
NTC	No-Template Control
VCF	Variant Call Format
CNS	Central Nervous System
PCA	Principal Component Analysis
EUR	European
AFR	African
EAS	East Asian
ARI	Adjusted Rand Index
NMI	Normalized Mutual Information
F_ST_	Fixation Index
IGA	Individual Genetic Ancestry
