# Supplementary material for: Population Genetics of Pharmacogenetic Variants in a Greek Psychiatric Cohort of over 3000 Individuals
Source: Int J Mol Sci. 2025 Oct 11;26(20):9896. doi: 10.3390/ijms26209896 (PMC12564037; doi:10.3390/ijms26209896)
Supplement: Supplementary file 1 [file ijms-26-09896-s001.zip › ijms-3888373-supplementary.pdf]

Supplementary Material

Table S1. SNPs and genes included in the PGx-CNS panel.

SNP	Gene
rs1800497	*ANKK1, DRD2*
rs12248560	*CYP2C19*
rs28399504	*CYP2C19*
rs4244285	*CYP2C19*
rs4986893	*CYP2C19*
rs1057910	*CYP2C9*
rs1799853	*CYP2C9*
rs1065852	*CYP2D6*
rs28371725	*CYP2D6*
rs35742686	*CYP2D6*
rs3892097	*CYP2D6*
rs5030655	*CYP2D6*
rs5030656	*CYP2D6*
rs1799978	*DRD2*
rs963468	*DRD3*
rs1051740	*EPHX1*
rs2234922	*EPHX1*
rs4713916	*FKBP5*
rs2832407	*GRIK1*
rs1414334	*HTR2C*
rs17782313	*MC4R*
rs489693	*MC4R*
rs3812718	*SCN1A*
rs7668258	*UGT2B7*
